# To Buy or Not to Buy Under Brand Image Transgression? Consumer Behavior and Underlying Mechanisms from a Power Distance Belief Perspective

**DOI:** 10.3390/bs16101863

**Published:** 2026-10-09

**Authors:** Lina Zhang, Bing Shi

**Affiliations:** Department of Psychology, Sun Yat-sen University, Guangzhou 510006, China; 18011714936@163.com

**Keywords:** power distance belief, brand image transgression, face consciousness, purchase intention, consumer behavior

## Abstract

Brand image transgression occurs when a brand-caused change in a brand’s current image, relative to its original image, creates a marked incongruence between consumers’ self-image and the brand image—a change that is negative for the brand’s original customer base. Compared with quality and moral transgressions, this covert type of brand transgression has received little scholarly attention, particularly with respect to individual differences in cultural orientation. Drawing on power distance belief (PDB), five experiments (total valid *N* = 1184) examined how consumers with different levels of PDB respond to brand image transgression, the mediating role of face consciousness, and the boundary condition of public versus private usage context. Under image transgression (vs. no transgression), the decline in purchase intention was significantly greater among high-PDB than among low-PDB consumers (Experiments 1 and 2). PDB was positively related to face consciousness, both correlationally and causally (Experiment 3). The results were consistent with face consciousness mediating the interactive effect of PDB and image transgression on purchase intention, whereas neither perceived quality risk nor perceived psychological risk showed such an effect (Experiment 4). The effect emerged in public usage contexts but was not statistically significant in private contexts (Experiment 5). These findings extend PDB research to the brand transgression domain, provide evidence consistent with face consciousness as an important mediating mechanism underlying consumer responses to image transgression, and offer guidance for transgression-recovery strategies tailored to consumers’ individual-level cultural orientations.

## 1. Introduction

As consumers have become increasingly aware of their rights and as social media has amplified their voice, brand-related transgressions can now be disseminated almost instantly by consumers themselves. Brand transgressions are no longer incidents that firms can fully avert through internal management or control, and enterprises must treat them with due seriousness (Xu et al., 2021). Such transgressions harm consumers’ interests and erode their trust and confidence in the firm; the resulting negative brand relationships can cost a brand many existing and potential customers and reduce consumer satisfaction, trust, and loyalty (Y. Xie & Peng, 2009). Brands should therefore strive to prevent transgression incidents and, once a transgression arises, respond promptly and effectively to mitigate its adverse impact (Joireman et al., 2016).

The consequences of a brand transgression depend closely on how consumers perceive and react to the incident. After the Volkswagen “Dieselgate” scandal broke in 2015, consumers in European and American markets reportedly received substantial compensation for the same fraudulent conduct—circumventing environmental emissions testing—whereas Volkswagen conducted no comparable recall or compensation program in the Chinese market, and its sales in China suffered no evident setback. Such starkly different reactions suggest that consumers may differ substantially in their perceptions of, and attitudes toward, the same brand transgression. Consumers are individuals embedded in social contexts who carry distinct sociocultural characteristics, and such differences need not coincide with national or cultural boundaries. This research therefore examines the role played in this process by an important individual-level cultural orientation: power distance belief (PDB), the degree to which people expect and accept disparities in power (Hofstede, 2001). Prior research on PDB in brand transgression contexts has found that when a brand is implicated in moral violations, such as encroaching on employees’ rightful benefits, consumers lower in PDB sympathize with the victims more than consumers higher in PDB do, which intensifies perceived harm and negative moral emotions and thereby produces more unfavorable responses toward the transgressing company (Xu et al., 2021).

Brand transgression incidents come in different types, chiefly image transgressions, quality transgressions, and moral transgressions (Fetscherin & Sampedro, 2019). Image transgressions involve changes to a brand’s existing image such that purchasing or using the brand damages consumers’ self-image. Compared with quality and moral transgressions, the harm inflicted by image transgressions is more indirect, and the damage to consumers or related groups is more covert and less easily detected. Yet in an environment where social media is pervasive and information spreads widely and rapidly, damage to one’s personal image can likewise produce substantial losses. To date, research on brand transgressions has concentrated largely on quality transgressions (Cleeren et al., 2017; Dawar & Pillutla, 2000; Vassilikopoulou et al., 2011) and moral transgressions (Dutta & Pullig, 2011; Xu et al., 2021), while brand image transgression continues to generate a large number of unresolved real-world problems. Premium automakers such as Mercedes-Benz, BMW, and Audi have diluted their premium positioning through repeated price cuts and lost part of their core customer base; smartphone brands have eroded the trust of their core fans through steep short-term price reductions; and DJI’s price cuts on professional product lines have driven away professional users. These cases collectively indicate that image transgression—a covert yet highly consequential category of brand transgression—can no longer be ignored, and yet dedicated research on its underlying psychological mechanisms remains scarce. This gap is precisely the entry point of the present research. What psychological mechanism drives consumer reactions to brand image transgression, and why do consumers with different levels of power distance belief react differently? Drawing on power distance belief, this research investigates changes in consumer behavior under brand image transgression and the psychological mechanism underlying them. The findings enrich research on how brand image transgression and power distance belief affect consumer behavior and offer brands practical guidance for formulating targeted transgression-recovery strategies based on consumer characteristics.

## 2. Theoretical Background

### 2.1. Brand Image Transgression

A transgression is the violation of some implicit or explicit rule (Aaker et al., 2004; Metts, 1994). A brand transgression is a brand-related crisis triggered by poor brand management (Tsarenko & Tojib, 2015) and can be classified into three types: quality transgressions, which concern product defects or quality problems; moral transgressions, which concern violations of the social values or ethics that the brand projects (Dutta & Pullig, 2011); and image transgressions, which concern a mismatch between the brand image and consumers’ self-image (Fetscherin & Sampedro, 2019).

Brand image has been defined as “the total perception of a brand—a set of beliefs that form a complete picture of the brand, shaped by information about the brand and past experience with it” (Assael, 1987). Brand image is a core element of brand management: firms design and position products and endow brands with particular images precisely to attract consumers’ brand choices. Brand consumption satisfies important psychological needs, such as constructing, expressing, and reinforcing self-identity and highlighting one’s distinctiveness from others (Sirgy, 1982). Purchasing and using particular brands is one way consumers signal to others that they conduct themselves appropriately and project a suitable image in specific situations (Back, 2005).

Whether a brand’s image is congruent with the consumer’s own image significantly influences consumers’ attitudes and intentions toward a product or service (Sirgy et al., 2000), and consumers are more inclined to purchase products that match their self-image (Aguirre-Rodriguez et al., 2012). Purchase intention is also significantly affected by social influence and brand image (Liang et al., 2024). Self-image congruence can explain and predict consumers’ product choice, brand choice, brand attitudes, purchase intention, store loyalty, and store preference (Sirgy et al., 1997). At the same time, consumers construct and develop their self-concept by disidentifying with brands that do not fit their own image (M. S. W. Lee et al., 2009), and individuals deliberately refrain from choosing certain brands partly to construct their self-concept and personal identity (Escalas & Bettman, 2005). Choosing brands that match one’s self-image and rejecting brands that do not are thus complementary ways in which consumers construct their self-concept and maintain self-image congruence. Related work shows that negative brand publicity affects consumers’ brand trust through self-congruence (Tong et al., 2023) and that a strong brand image helps alleviate the consumer uncertainty created by stereotypes (Islam & Hussain, 2023).

Prior research indicates that congruence and incongruence alike involve the positive or negative valence of self-image and product image (Sirgy, 1982): positive congruence is a match between a positive product-image perception and a positive self-image belief, whereas negative congruence is a match between a negative product-image perception and a negative self-image belief. Correspondingly, incongruence between brand image and consumer image takes two forms: positive incongruence refers to a relatively positive product-image perception paired with a negative consumer self-image, whereas negative incongruence refers to a relatively negative product-image perception paired with a positive consumer self-image. Purchase motivation is strongest under positive congruence, followed in descending order by positive incongruence, negative congruence, and negative incongruence (Sirgy, 1982). Brand image transgression primarily involves negative incongruence, that is, incongruence between a relatively negative product image and a positive consumer self-image. Importantly, negative image incongruence can arise in various real-world situations, not all of which constitute image transgression; the essential distinction is whether the incongruence is caused by the brand itself. As consumers progress through their life cycle, for example, the brand image of a given period may no longer fit their current self-image; this incongruence is not created by the brand but stems from consumers’ own development or life-cycle changes (Fetscherin & Sampedro, 2019). Likewise, individuals may avoid a brand because it represents an undesired self they wish to distance themselves from, or a negative reference group with which they do not want to be associated (M. S. W. Lee et al., 2009); this incongruence, too, is triggered by consumers’ past experiences or cognitions rather than by the brand. Although both situations fall within the scope of image incongruence, neither constitutes a brand image transgression.

Accordingly, this research defines brand image transgression as follows: owing to causes attributable to the brand, the brand’s current image changes relative to its original image, thereby producing a marked incongruence between consumers’ self-image and the brand image; when this incongruence-inducing change is negative for the brand’s original consumer group, it constitutes an image transgression.

### 2.2. Power Distance Belief

This research focuses on the role of power distance belief when consumers confront brand image transgressions. Power distance belief refers to the degree to which people expect and accept disparities in power (Hofstede, 1984, 2001; Oyserman, 2006). It is important to distinguish two levels at which power distance has been conceptualized: the national or cultural level (power distance) and the individual level (power distance belief). These two levels exert analogous influences on consumer behavior, but they are not interchangeable, and the present research examines PDB exclusively at the individual level. The culture-as-situated-cognition model (Oyserman & Lee, 2007; Y. Zhang et al., 2010) provides the theoretical basis for this individual-level focus. This framework treats culture as a form of situated cognition and holds that culture shapes the content of cognition (what), the processes of cognition (how), and motivation (for what purposes). It further proposes that individuals within the same national culture can develop different cultural orientations through learning and other mechanisms, and that priming culture-related content significantly shifts the accessibility of cultural knowledge, values, self-descriptions, and understandings of one’s social obligations and relationships with others (Oyserman & Lee, 2007). Consistent with this framework, individuals learn cultural values such as PDB, as well as related concepts such as self-control, through various mechanisms, forming an associative network of culture-related concepts: in high-PDB contexts, people acquire self-concepts such as self-restraint and self-control and tend to conform to socially approved behavior when facing social taboos or socially disapproved situations, whereas in low-PDB contexts such notions of restraint or alignment with social expectations are absent (Oyserman & Lee, 2007). A systematic review of nearly two decades of PDB research in the consumer domain has further clarified the activation and measurement of PDB at the individual level and provides an integrative framework for subsequent research (Fan & Zhang, 2022). PDB does not measure a person’s actual level of power (Oyserman, 2006); rather, it captures the subjective degree to which a person expects and accepts inequality. The core difference between individuals high and low in PDB lies in their attitudes toward power disparities. Individuals high in PDB regard social hierarchy as acceptable and wish to maintain important social hierarchies; individuals low in PDB regard social equality as essential and wish to promote more equitable outcomes. People higher in PDB view inequality as a fact of life to which one must adapt, whereas people lower in PDB believe that all people are inherently of equal worth and regard inequality as illegitimate (Oyserman, 2006; Y. Zhang et al., 2010). Throughout this manuscript, “high PDB” and “low PDB” therefore refer to individuals who differ in their endorsement of power disparities, not to national or cultural categories.

Different levels of PDB (high vs. low) have been shown to influence consumer behavior in numerous domains, including charitable giving (Winterich & Zhang, 2014), price–quality judgments and preferences for user-designed products (Paharia & Swaminathan, 2019), reactions to corporate moral transgressions (Xu et al., 2021), preferences for celebrity-endorsed restaurants (Huo et al., 2022), preferences for anthropomorphized digital advertising (van Esch et al., 2023), consumer purchase avoidance (H. Lee & Lalwani, 2024), and perceptions of the service fairness of human versus AI frontline agents (Wu et al., 2024). Building on these findings, this research explores the role that consumers’ power distance belief may play in the domain of brand image transgression.

## 3. Hypothesis Development

### 3.1. Power Distance Belief and Brand Image Transgression

First, differences in power distance belief lead consumers to differ in their concern for social status. Numerous studies have shown that high-power-distance leaders tend to focus on power and status, believing that they should enjoy privileges and authority within the organization, whereas low-power-distance managers regard the distinction between themselves and their subordinates as a matter of organizational division of labor rather than of status (Kirkman et al., 2009). Consumers with high PDB hold a stronger status-oriented mindset (Kim & Zhang, 2014); they regard status products as effective symbols of social status (Gao et al., 2016) and are more eager to enhance their own social status (Eastman et al., 1999). High-PDB consumers (relative to low-PDB consumers) are more inclined to engage in status-signaling behaviors, such as consuming luxury goods and other material products to display their superior standing (Gao et al., 2016; Wang et al., 2020). Under high PDB, consumers expect to use products to enhance or maintain their social status (Belk, 1988; Hyun & Park, 2016). Regarding whether consumers prefer brands designed by an authoritative company or brands designed by users, consumers with high PDB prefer company-designed products, whereas consumers with low PDB prefer user-designed brands (Paharia & Swaminathan, 2019). Research on the interactive influence of PDB and consumer status on preference for domestic brands likewise shows that under high PDB, low-status (relative to high-status) consumers are more easily attracted to domestic brands (Wang et al., 2020). These studies consistently indicate that high-power-distance individuals attach greater importance to social status than low-power-distance individuals do.

Second, differences in power distance beliefs lead consumers to hold different attitudes toward social approval. Under high PDB, individuals confronted with social taboos or socially disapproved situations conform to what is socially approved so as to remain consistent with others; under low PDB, no such notions of self-restraint or alignment with social expectations prevail (Oyserman & Lee, 2008). Cross-national evidence likewise shows that in high-power-distance countries restraint is especially encouraged in the face of socially prohibited temptations and students tend not to challenge their teachers face-to-face, whereas in low-power-distance countries restraint is not similarly encouraged and students are encouraged to voice dissenting opinions openly (Y. Zhang et al., 2010); the same logic applies at the individual level, where people higher (vs. lower) in PDB are more accepting of hierarchical arrangements. In low-power-distance environments, employees may express their views directly to their bosses (Kirkman et al., 2009); by contrast, high-power-distance individuals more readily accept power inequality between superiors and subordinates, tend to follow formal codes of conduct, and are unwilling to disagree with their superiors (Hofstede, 1983). People with a high power distance orientation tend to behave deferentially in front of managers, avoid disagreement, regard bypassing their boss as insubordination, and believe that leaders deserve respect and esteem (Hofstede, 2001). Individuals high in PDB also exhibit less impulsive buying because their self-control is stronger than that of individuals low in PDB (Y. Zhang et al., 2010). These studies consistently indicate that high-power-distance individuals place greater value on conforming to social approval than low-power-distance individuals do.

Taken together, when a brand image transgression occurs, purchasing the brand’s products conflicts with high-PDB consumers’ need to maintain or enhance their social status—the original brand image can no longer represent that status—so their purchase intention is likely to fall. Moreover, to remain consistent with others, maintain social approval, and avoid violating social taboos, high-PDB consumers’ purchase intention may decline further still. By contrast, low-PDB consumers do not purchase products primarily to enhance or maintain social status; their purchases rest more on the product itself. An image transgression should therefore have little effect on low-PDB consumers’ self-identity or self-expression, and any decline in their purchase intention should be comparatively small. On this basis, we propose:

**Hypothesis** **1** **(H1).** *Under high power distance belief, consumers’ purchase intention is significantly lower under image transgression than under no transgression; under low power distance belief, purchase intention does not differ significantly between the two conditions*.

### 3.2. Power Distance Belief and Face Consciousness

The differing purchase intentions of consumers with different power distance beliefs under image transgression may stem from their differing face consciousness. “Face” refers to the face an individual expects to possess in society; it represents a pleasant sense of social self-worth that a person expects others to acknowledge (J. Li et al., 2015). Face is a social attribute and public image acquired through interaction with others—a social image in consumers’ minds that reflects how they view themselves when interacting with others (J. Li et al., 2015). It is the positive social image and social perception that an individual expects to possess in interpersonal interactions, and it is dynamic in nature: it may strengthen, weaken, or remain stable over time. Face consciousness denotes the positive image and positive value that individuals seek to display through their own efforts in interpersonal interactions in order to be recognized by others (Goffman, 1955).

Face consciousness exhibits cultural differences. A study of the consumption behavior of college students in China and the United States found that Chinese consumers are more face-conscious than American consumers (Bao et al., 2003). Asian consumers remain keen on luxury consumption despite relatively low income levels because Chinese consumers are more susceptible to prestige motives than American consumers (J. J. Li & Su, 2007). Face and status-need motives shape the consumption preferences of Chinese wine consumers, who prefer cork-stoppered rather than screw-cap bottles because, in Chinese culture, cork closures more readily evoke associations with prestige, face, and status (Somogyi et al., 2011). One study found that when Chinese participants experienced transient financial deprivation, their prosocial behavior increased through face consciousness, demonstrating the importance of face consciousness in the economic-deprivation effect among Chinese people (S. Xie & Shi, 2022).

In high-PDB environments, the social hierarchical order is clearly defined and interpersonal interaction emphasizes face. In social environments with high face consciousness, people are taught to attend to face (Bao et al., 2003)—not only their own but also that of others—and in their mutual interactions they seek to avoid “hurting harmony and face”; face consciousness is therefore generally strong. In low-PDB environments, by contrast, people are less accepting of unequal power distribution and are committed to maintaining an egalitarian social order: employees may voice their views directly to their bosses (Kirkman et al., 2009), and students may openly raise dissenting opinions with their teachers in class (Y. Zhang et al., 2010). They do not suppress their true views to preserve face, so face consciousness is generally weak. A relationship may thus exist between power distance belief and face consciousness. Given these differences between individuals with different levels of PDB and the characteristics of face consciousness, we hypothesize:

**Hypothesis** **2** **(H2).** *Consumers with high (vs. low) power distance belief have stronger face consciousness*.

### 3.3. The Mediating Role of Face Consciousness

Face consciousness is closely related to power distance belief. People generally expect to maintain and reinforce their self-concept, and purchasing and using products is an important means to this end (Graeff, 1996). Purchasing and using particular brands is one way for consumers to signal to others that they behave appropriately and project a suitable image in specific situations (Back, 2005). Individuals in high-power-distance environments are more inclined than those in low-power-distance environments to choose status goods (Kim & Zhang, 2014), and high-PDB consumers expect to use products to enhance or maintain their social status (Belk, 1988; Hyun & Park, 2016). In parallel, consumers with strong face consciousness are more inclined to purchase branded products to satisfy their social needs and display their wealth to others (Liao & Wang, 2009). Consumers with high face consciousness pay more attention to the external attributes of products or services (e.g., brand, social renown) than to internal attributes (e.g., quality); they hope to elevate their perceived social status by displaying status, prestige, and self-image to others (Belk, 1988; Hyun & Park, 2016), which is consistent with high-PDB consumers’ stronger preference for status products. In other words, when deciding whether to purchase a product, consumers with high face consciousness weigh whether the purchase will enhance or diminish their image and status; they give greater weight to the social impact of the product—whether buying or not buying it will gain or lose them face. In transgression contexts, high-power-distance individuals are more sensitive to status signals (Gao et al., 2016); the “devaluation” of the brand image caused by a transgression directly damages the status signal they convey through the brand, their perception of face loss is stronger, and their purchase intention therefore declines more markedly.

Face consciousness is also closely tied to consumers’ social image. Because of face, Chinese people care more about how others view them and are more prone than Westerners to feel anxious about their image in public settings. For consumers with high face consciousness, social self-image exerts a pronounced influence on their behavioral intention to purchase products (Kaur & Soch, 2018). Because the desire to gain face aims to enhance the perceived social self, such consumers may purchase a product to enhance face even when the product is unrelated to face in the eyes of others. Consumers with high face consciousness care more about how others see them, whether they can win others’ approval, and how others evaluate them (Wong & Ahuvia, 1998). For consumers with low face consciousness, by contrast, social self-image has little influence on behavioral intention to purchase, because such consumers place greater value on their actual personal image than on showing off to others (Kaur & Soch, 2018). Indian consumers, for example, value face more than Canadian consumers do—their face consciousness is stronger, and they attach greater importance to their social image—whereas Canadian consumers focus more on their personal image than on their social image when making smartphone purchase decisions (Kaur & Soch, 2018). Face consciousness is thus inseparably linked to social image.

Based on the above, we propose:

**Hypothesis** **3** **(H3).** *The interaction effect proposed in H1 is mediated by face consciousness: under image transgression (vs. no transgression), high power distance belief consumers experience stronger face consciousness, which in turn produces a more pronounced decline in purchase intention than that of low power distance belief consumers*.

### 3.4. The Moderating Role of Public Versus Private Usage Context

Visibility in the course of product consumption is an important factor influencing consumer behavior. Visible products and services serve as bases for inferring the status, personality, and character of their owners or consumers (Belk, 1978). Perceptions of visibility can vary considerably as a function of product type, consumption situation, usage conditions, product-related topics, and individual-difference variables (Chuah et al., 2016). Participants are more likely to donate to charity in public settings than in private settings (Roux et al., 2015). People experiencing transient economic deprivation behave more prosocially, and this increase in prosocial behavior is observed in public settings but diminishes in private settings (S. Xie & Shi, 2022). When consuming publicly consumed products, people are more likely to follow what they believe others will like, regardless of their own personal preferences (Bearden & Etzel, 1982). When products are used in public or the behavior takes place in public settings, Chinese consumers are typically willing to spend more than when the product or service is used privately or at home (Lin et al., 2013). One study found that high-PDB consumers were interested in purchasing a luxury handbag only when it would be publicly displayed to others; when the handbag was to be used in a private setting, their purchase intention was lower (Gao et al., 2016). Therefore, depending on whether an image-transgressing product is used in a public or a private context, the purchase intentions of high- (vs. low-) PDB consumers may differ significantly. In public contexts, an image-transgressing product threatens consumers’ social self-image, thereby lowering their purchase intention. We thus propose:

**Hypothesis** **4** **(H4).** *The effect of image transgression on the purchase intention of consumers with different power distance beliefs is moderated by public versus private usage context. In public contexts, the decline in purchase intention toward an image-transgressing brand (vs. a non-transgressing brand) is more pronounced for high power distance belief consumers than for low power distance belief consumers; in private contexts, the difference between the two groups narrows and is expected to be no longer statistically significant*.

### 3.5. Overview of the Present Research

**Figure 1:**
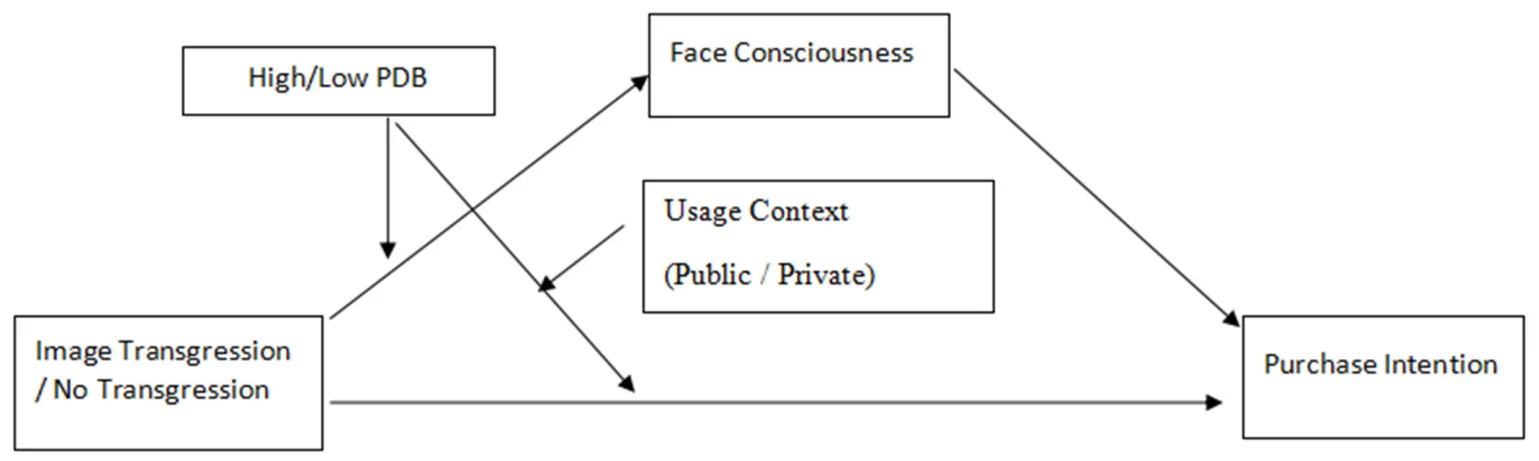
Conceptual framework of the present research.

This research centers on the mechanism through which power distance belief operates under brand image transgression and tests the theoretical hypotheses progressively across five experiments. The design is hierarchical: we first test the first-order moderating role of power distance belief in the effect of image transgression on purchase intention, and on this basis examine usage context as a higher-order boundary condition of the PDB × image transgression interaction. Experiments 1 and 2 test H1 (under image transgression, the decline in purchase intention is significantly greater for high-PDB than for low-PDB consumers). Experiment 3 tests H2 (high-PDB consumers have stronger face consciousness). Experiment 4 tests H3 (face consciousness mediates the interactive effect of PDB and image transgression on purchase intention). Experiment 5 tests H4 (public versus private usage context moderates the effect: in public contexts, the decline in purchase intention is more pronounced for high-PDB consumers, whereas in private contexts the difference is expected to be no longer statistically significant). Figure 1 presents the conceptual framework, and Table 1 summarizes the five experiments.

## 4. Pretests

### 4.1. Selection of the Power Distance Belief Scale

Before the formal experiments, a pretest evaluated candidate measurement scales for power distance belief. Three PDB scales are in common use. The first is a nine-item scale (Neuliep, 2015) (five-point; 1 = “strongly disagree,” 5 = “strongly agree”), with items such as “In an organization, employees should feel comfortable expressing disagreement to their managers” and “In a classroom, students should be allowed to express their own views on a topic without being punished by the teacher or professor”; items 1, 2, 3, 6, and 9 are reverse-coded. A pretest with 299 participants indicated poor model fit (RMSEA = 0.178, CFI = 0.770, TLI = 0.694, SRMR = 0.110) and modest internal consistency (α = 0.741), so this scale was not adopted. The second is an eight-item scale (Y. Zhang et al., 2010) (seven-point; 1 = “strongly disagree,” 7 = “strongly agree”), with items such as “As citizens, we should attach great importance to conformity” and “It is good to be able to work with a manager who gives subordinates the reasons for his decisions and answers any questions they may raise”; items 2, 3, 7, and 8 are reverse-coded. A pretest with 138 participants indicated poor model fit (RMSEA = 0.217, CFI = 0.444, TLI = 0.221, SRMR = 0.150) and low internal consistency (α = 0.414), so this scale was not adopted either. The third is a five-item scale (Yoo et al., 2011) (seven-point; 1 = “strongly disagree,” 7 = “strongly agree”), with items such as “People in higher positions should make most decisions without consulting people in lower positions” and “People in higher positions should not ask the opinions of people in lower positions too frequently.” A pretest with 154 participants submitted the five items to confirmatory factor analysis (CFA); model fit was good (RMSEA = 0.060, CFI = 0.990, TLI = 0.980, SRMR = 0.030) and internal consistency was high (α = 0.819).

Given its superior reliability and validity relative to the first two scales, and considering the substantive meaning of its items, we adopted the third scale (Yoo et al., 2011) both to measure power distance belief directly and to serve as the manipulation check for power distance belief in the experiments.

### 4.2. Development of the Image-Transgression Material

In the experiments, the image-transgression and no-transgression conditions were induced through reading materials. Because real brands carry numerous confounding associations, a fictitious sneaker brand, TANIC, was used. As prior experimental research offered no existing materials on brand image transgression, the transgression scenario was designed from the construct definition itself. An initial version read, in part: “the brand has changed designers; this year’s new designs are hardly different from those on a certain group-buying website; this year’s styles clearly feel less satisfying and less expressive of your individuality.” In a pretest (*N* = 89), 32.6% of participants classified this material as a quality transgression, 7.9% as a moral transgression, and 59.6% as an image transgression. A goodness-of-fit chi-square test with the expected proportion set at 0.85 for the image-transgression option (and 0.15 for the other two options combined) was not significant, χ^2^ = 3.047, *p* = 0.218; the material thus failed to differentiate the three transgression types adequately. Additional brand-transgression scenarios were therefore collected and evaluated, and the following scenario was ultimately adopted: “on a high-speed train, you see a group of middle-aged men and women, loudly playing videos on their phones, who are also wearing the brand.” In a further pretest of the revised material (*N* = 47), 97.87% of participants classified the material as an image transgression, one participant classified it as a moral transgression, and none classified it as a quality transgression; the classification rate significantly exceeded the preset 0.85 criterion, χ^2^ = 6.108, *p* < 0.05. The revised scenario was therefore adopted. It should be noted that this manipulation operationalizes one specific manifestation of image transgression—brand-image dilution involving loss of exclusivity and association with disliked users—rather than the full breadth of possible image transgressions; this scope condition is revisited in the General Discussion. The full image-transgression material appears in Appendix A.1.

### 4.3. Validation of the Reading Materials

The no-transgression material described the same TANIC brand with identical design, quality, and popularity information but without any transgression content (see Appendix A.2). A further pretest compared the two materials. Eighty-eight participants were randomly assigned to the image-transgression condition, of whom 4 failed the reading-material check questions, leaving 84 valid participants; 66 participants were randomly assigned to the no-transgression condition, of whom 10 failed the check questions, leaving 56 valid participants. The manipulation check employed a six-item self-image congruence scale (Sirgy et al., 1997) (α = 0.907), with items such as “The image of the typical user of the sneakers is similar to how other people see me”; scores were averaged across the six items. Participants in the image-transgression condition (*M* = 3.26, *SD* = 1.34) reported significantly lower self-image congruence than those in the no-transgression condition (*M* = 3.76, *SD* = 1.09), *t*(138) = 2.42, *p* < 0.05, indicating that the reading materials effectively differentiated the image-transgression condition from the no-transgression condition.

## 5. Experiment 1: Purchase Intention of Consumers with Different Power Distance Beliefs Under Image Transgression

Experiment 1 manipulated the two core variables—power distance belief and brand image transgression—to provide an initial test of H1, namely that under high PDB, purchase intention is significantly lower under image transgression than under no transgression, whereas under low PDB the two conditions do not differ. The experiment employed a dual-priming paradigm to control for interference from individuals’ chronic power distance beliefs and used manipulation checks to verify the validity of both manipulations, thereby establishing the basic effect for the subsequent experiments on mechanism and boundary conditions.

### 5.1. Method

A 2 (power distance belief: high vs. low) × 2 (transgression: image transgression vs. no transgression) between-subjects design was adopted. The required sample size was calculated with G*Power 3.1.9.6. (Faul et al., 2009); for a four-group ANOVA with effect size *f* = 0.40, α = 0.05, and power = 0.80, the minimum required sample size was 73. Participants were recruited through a relevant elective course and received course credit after completing the questionnaire. A total of 278 participants were recruited; 3 failed the attention-check question and 4 did not complete the power distance belief priming task, leaving 271 valid participants (44% male; aged 18–22 years, *M*_age_ = 19.17, *SD* = 0.93).

Participants were first randomly assigned to either the high- or the low-PDB priming condition. To prime power distance belief, a scrambled-sentence task was used (Oyserman & Lee, 2008; Y. Zhang et al., 2010). In the high-PDB condition, participants unscrambled and assembled ten word sets related to power-hierarchy inequality (e.g., “social hierarchy/necessary/is”); in the low-PDB condition, participants unscrambled ten word sets related to power-hierarchy equality (e.g., “social hierarchy/unnecessary/is”). To strengthen the priming, participants in the high-PDB condition then wrote three reasons supporting the power distance proposition “There is an order of inequality in this world in which everyone has a rightful place, and both high and low positions should be protected by this order,” whereas participants in the low-PDB condition wrote three reasons opposing it (Y. Zhang et al., 2010). After the priming, participants completed the five power distance belief items (Yoo et al., 2011) (α = 0.797), averaged to form a PDB score. Next, participants read the image-transgression (or no-transgression) material; the manipulation check used the six-item self-image congruence scale (Sirgy et al., 1997) (α = 0.916), averaged to form a self-image congruence score. Purchase intention was then measured with a four-item scale (Huang & Deng, 2022) (α = 0.932), with items such as “You would be willing to purchase TANIC sneakers.” Finally, demographic information was collected.

### 5.2. Results

Manipulation checks. Independent-samples *t* tests showed that participants in the high-PDB condition (*M* = 2.79, *SD* = 1.03) scored higher on PDB than those in the low-PDB condition (*M* = 2.53, *SD* = 0.99), *t*(269) = 2.07, *p* < 0.05, indicating that the priming of power distance belief was successful. Participants in the image-transgression condition (*M* = 3.09, *SD* = 1.15) scored lower on self-image congruence than those in the no-transgression condition (*M* = 3.49, *SD* = 1.30), *t*(269) = 2.68, *p* < 0.05, indicating that the manipulation of image transgression was successful.

Purchase intention. A two-way ANOVA with power distance belief (high vs. low) and transgression condition (image transgression vs. no transgression) as independent variables and purchase intention as the dependent variable revealed no significant main effect of power distance belief, *F*(1, 267) = 0.18, *p* = 0.67, and no significant main effect of transgression condition, *F*(1, 267) = 1.29, *p* = 0.26. The interaction between power distance belief and transgression condition was significant, *F*(1, 267) = 4.73, *p* = 0.03, η_p_^2^ = 0.02: under high PDB, participants in the image-transgression condition (*M* = 4.16, *SD* = 1.29) reported lower purchase intention than those in the no-transgression condition (*M* = 4.31, *SD* = 0.97), whereas under low PDB, participants in the image-transgression condition (*M* = 4.41, *SD* = 1.07) reported higher purchase intention than those in the no-transgression condition (*M* = 3.93, *SD* = 1.36). H1 thus received preliminary support.

### 5.3. Discussion

Using dual priming of power distance belief and image transgression, Experiment 1 provided initial evidence that under brand image transgression (vs. no transgression), the decline in purchase intention is significantly greater for high-PDB than for low-PDB consumers. The manipulation checks indicated that both the priming of power distance belief and the manipulation of image transgression were effective, strengthening the internal validity of this conclusion.

However, Experiment 1 had a noteworthy limitation in its materials: the no-transgression material did not include a “discount promotion” description matching that of the transgression material. The image-transgression material explicitly mentioned that the brand had adopted a discounting strategy in response to an economic downturn, leading large numbers of non-target consumers to purchase the brand, whereas the no-transgression material described only the brand’s positive image and positioning without promotional information of comparable magnitude. This asymmetry may have allowed the “discount promotion” element to exert an additional positive influence on low-PDB participants in the transgression condition, producing the observed rise in their purchase intention rather than an effect driven purely by the image transgression itself. To address this potential confound, a discount scenario was added to the no-transgression material, and Experiment 2 re-examined the effect under more rigorous control.

## 6. Experiment 2: Controlling for the Price-Promotion Confound

Experiment 1 provided initial evidence for the differential effect of power distance belief on purchase intention under image transgression, but the unmatched “discount” content left a price-promotion confound as an alternative explanation for the increased purchase intention observed in the low-PDB transgression group. Experiment 2 therefore added discount-promotion information to the no-transgression material, consistent with the transgression material, and replicated the test of H1 under more rigorous experimental control.

### 6.1. Method

A 2 (power distance belief: high vs. low) × 2 (transgression: image transgression vs. no transgression) between-subjects design was adopted. The required sample size was calculated with G*Power 3.1 (Faul et al., 2009); for a four-group ANOVA with effect size *f* = 0.40, α = 0.05, and power = 0.80, the minimum required sample size was 73. Participants were recruited through a relevant elective course and received course credit after completing the questionnaire. A total of 135 participants were recruited. Of these, 14 failed the reading-material check questions, 2 were excluded for reporting never having worn sneakers, 2 were excluded for careless responding (selecting the first option on 10 consecutive items), and 2 were excluded because their response times exceeded ±3 standard deviations, leaving 115 valid participants (27% male; aged 18–22 years, *M*_age_ = 19.16, *SD* = 0.72).

Participants were first randomly assigned to the high- or low-PDB priming condition; the priming procedure was identical to that of Experiment 1, after which participants completed the power distance belief items (Yoo et al., 2011) (α = 0.879). Participants then read the image-transgression (or no-transgression) material. The image-transgression material was identical to that of Experiment 1, whereas the no-transgression material now included a discount scenario of magnitude comparable to the transgression material (see Appendix A.3). The manipulation check used the self-image congruence scale from Experiment 1 (Sirgy et al., 1997) (α = 0.901), after which purchase intention (Huang & Deng, 2022) (α = 0.914) and demographic information were collected.

### 6.2. Results

Manipulation checks. Independent-samples *t* tests showed that participants in the high-PDB condition (*M* = 2.90, *SD* = 1.33) scored higher on PDB than those in the low-PDB condition (*M* = 2.24, *SD* = 1.01), *t*(113) = 2.98, *p* < 0.05, indicating successful priming. Participants in the image-transgression condition (*M* = 2.96, *SD* = 1.09) scored lower on self-image congruence than those in the no-transgression condition (*M* = 3.71, *SD* = 1.26), *t*(113) = 3.41, *p* < 0.05, indicating a successful transgression manipulation.

**Figure 2:**
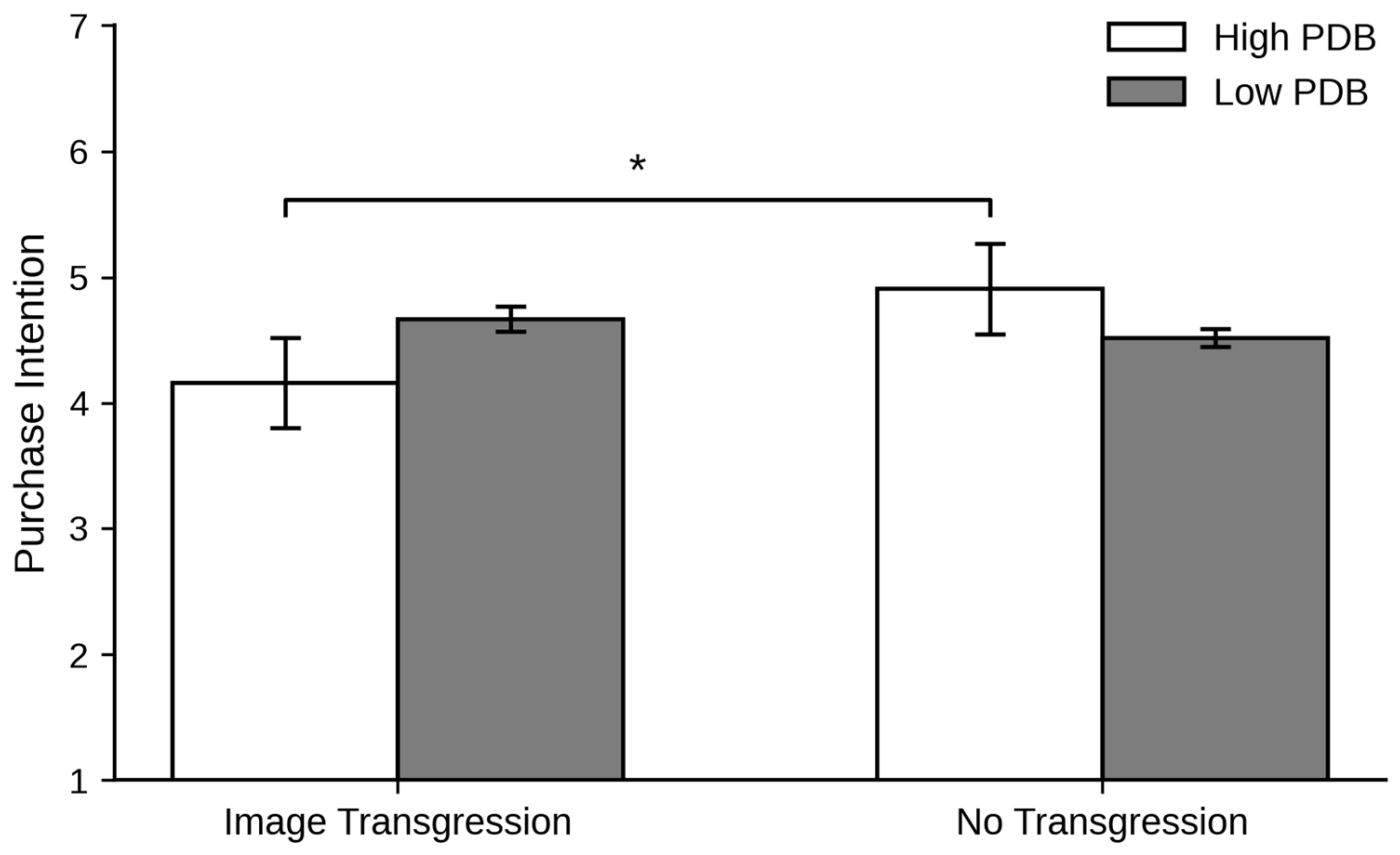
Purchase intention of consumers with different power distance beliefs under image transgression (Experiment 2). Error bars represent ±1 *SE*. * *p* < 0.05.

Purchase intention. A two-way ANOVA with power distance belief and transgression condition as independent variables and purchase intention as the dependent variable revealed no significant main effect of power distance belief, *F*(1, 111) = 0.12, *p* = 0.74, and no significant main effect of transgression condition, *F*(1, 111) = 2.15, *p* = 0.12. The interaction was significant, *F*(1, 111) = 5.89, *p* = 0.017, η_p_^2^ = 0.05: under high PDB, participants in the image-transgression condition (*M* = 4.16, *SD* = 0.93) reported significantly lower purchase intention than those in the no-transgression condition (*M* = 4.91, *SD* = 1.17), *p* = 0.006, whereas under low PDB, purchase intention did not differ between the image-transgression condition (*M* = 4.67, *SD* = 0.82) and the no-transgression condition (*M* = 4.52, *SD* = 1.08), *p* = 0.539 (see Figure 2). H1 was thus further supported.

### 6.3. Discussion

With the “discount promotion” confound controlled, Experiment 2 again indicated that the effect of brand image transgression on purchase intention differs significantly as a function of power distance belief: high-PDB consumers showed a marked decline in purchase intention under image transgression, whereas low-PDB consumers showed no significant difference between conditions. This result replicates the core finding of Experiment 1 and, having controlled for the price-promotion account, further supports the robustness of H1.

Of particular note, once the “discount promotion” confound was controlled, the low-PDB group showed no statistically significant difference in purchase intention between the image-transgression and no-transgression conditions (*p* = 0.539), whereas the upward trend observed in Experiment 1 had suggested an increase. This contrast indicates that the earlier increase was attributable to the promotional information rather than to the image transgression itself, further attesting to the stability of the moderating role of power distance belief in responses to image transgression.

## 7. Experiment 3: The Relationship Between Power Distance Belief and Face Consciousness

Experiments 1 and 2 repeatedly verified the differential effect of power distance belief on purchase intention under image transgression, but the underlying mechanism remained to be established. Experiment 3 therefore comprised two progressive sub-studies. Experiment 3a measured participants’ chronic power distance belief and face consciousness to establish the correlation between the two constructs; Experiment 3b then manipulated power distance belief to examine its causal effect on face consciousness—that is, to test H2.

### 7.1. Experiment 3a

#### 7.1.1. Method

Experiment 3a measured power distance belief and face consciousness to test whether the two are correlated. The required sample size was calculated with G*Power 3.1 (Faul et al., 2009); for a correlation analysis with effect size ρ = 0.30, α = 0.05, and power = 0.80, the minimum required sample size was 82. Participants were recruited through relevant elective courses and received course credit upon completing the questionnaire. A total of 140 participants were collected, of whom 2 failed the attention-check question, leaving 138 valid participants (38% male; aged 18–22 years, *M*_age_ = 19.30, *SD* = 0.85).

Participants first completed the five-item power distance belief scale (Yoo et al., 2011) (α = 0.843), followed by a measure of face consciousness (X. Zhang, 2012) (α = 0.904). The face-consciousness questionnaire comprises eleven items distinguishing the desire to gain face from the fear of losing face: items 1–6 capture the desire to gain face (e.g., “I hope that others think I can accomplish things that ordinary people cannot”), and items 7–11 capture the fear of losing face (e.g., “Even when I genuinely do not understand something, I try my best to avoid appearing ignorant to others”). A separate pretest of this questionnaire with 154 participants (all valid) showed good CFA model fit (RMSEA = 0.079, CFI = 0.938, TLI = 0.914, SRMR = 0.070) and high internal consistency (α = 0.836).

#### 7.1.2. Results

A Pearson correlation analysis revealed a significant positive correlation between power distance belief and face consciousness (*r* = 0.280, *p* < 0.001): as power distance belief strengthens, face consciousness likewise tends to increase.

### 7.2. Experiment 3b

#### 7.2.1. Method

Building on the correlation established in Experiment 3a, Experiment 3b verified, through experimental manipulation, the difference in face consciousness between consumers with high versus low power distance belief. The required sample size was calculated with G*Power 3.1 (Faul et al., 2009); for an independent-samples *t* test with effect size *d* = 0.50, α = 0.05, and power = 0.80, the minimum required sample size was 128. Participants were recruited through elective courses and received course credit upon completing the questionnaire. A total of 185 participants were collected; 4 did not complete the scrambled-sentence priming task, leaving 181 valid participants (54% male; aged 18–21 years, *M*_age_ = 19.15, *SD* = 0.89).

Participants were first randomly assigned to the high- or low-PDB priming condition; the priming procedure was identical to that of Experiment 1, and participants then completed the power distance belief items (Yoo et al., 2011) (α = 0.797). Face consciousness (X. Zhang, 2012) (α = 0.849) was then measured, with scores averaged across the eleven items.

#### 7.2.2. Results

Manipulation check. An independent-samples *t* test showed that participants in the high-PDB condition (*M* = 2.87, *SD* = 1.03) scored higher on PDB than those in the low-PDB condition (*M* = 2.56, *SD* = 1.01), *t*(179) = 2.09, *p* < 0.05, indicating successful priming.

Face consciousness. Participants in the high-PDB condition (*M* = 4.20, *SD* = 0.91) scored significantly higher on face consciousness than those in the low-PDB condition (*M* = 3.93, *SD* = 0.86), *t*(179) = 1.99, *p* < 0.05, supporting H2: consumers with high (vs. low) power distance belief have stronger face consciousness.

### 7.3. Discussion

Through two progressive sub-studies, Experiment 3 systematically examined the intrinsic link between power distance belief and face consciousness, providing a key logical premise for uncovering the psychological mechanism at work under image transgression. At the trait level, Experiment 3a found a significant positive correlation between chronic power distance belief and face consciousness: the higher a consumer’s power distance belief, the stronger the face consciousness. Experiment 3b clarified the causal direction of this relationship through experimental manipulation: temporarily priming high power distance belief significantly increased face consciousness, directly supporting H2 and indicating that high power distance belief is an antecedent of heightened face consciousness. These results provide the foundation for the subsequent mediation test: given that heightened power distance belief strengthens face consciousness, and that brand image transgression damages precisely the social image consumers obtain through brand consumption, face consciousness may play an important mediating role linking power distance belief to changes in purchase intention under image transgression. Experiment 4 tests this prediction (H3) with a moderated mediation model.

## 8. Experiment 4: The Mediating Effect of Face Consciousness

Experiment 4 was designed to test the mediating effect of face consciousness (H3). Prior research has found that perceived risk mediates the relationship between quality transgression and purchase intention (Huang & Deng, 2022), whereas no research has examined whether perceived risk mediates the relationship between image transgression and purchase intention. To evaluate whether this alternative pathway could account for the effect, Experiment 4 additionally measured perceived quality risk and perceived psychological risk and tested their mediating effects in competition with face consciousness.

### 8.1. Method

A single-factor, two-level (image transgression vs. no transgression) between-subjects design was employed, with power distance belief measured. The required sample size was calculated with G*Power 3.1 (Faul et al., 2009); for a two-group one-way ANOVA with effect size *f* = 0.25, α = 0.05, and power = 0.80, the minimum required sample size was 128. Participants were recruited through relevant elective courses and received course credit upon completing the questionnaire. A total of 327 participants were collected. Of these, 43 failed the reading-material check question, 2 failed the attention-check question, 5 were excluded for reporting never having worn sneakers, 12 were excluded for careless responding (selecting the first option on 10 consecutive items), 1 was excluded as an aberrant case (a response time of 32,572 s), and 5 were excluded because their response times exceeded ±3 standard deviations. This left 260 valid participants (36% male; aged 18–21 years, *M*_age_ = 18.43, *SD* = 0.81).

Participants first completed the chronic power distance belief measure (Yoo et al., 2011) (α = 0.882). They were then randomly assigned to read the image-transgression or no-transgression material (identical to Experiment 2); the manipulation check used the self-image congruence scale (Sirgy et al., 1997) (α = 0.898). Purchase intention (Huang & Deng, 2022) (α = 0.903) and face consciousness (adapted from X. Zhang, 2012) were then measured, followed by perceived quality risk and perceived psychological risk (Laroche et al., 2004) and demographic information.

Face-consciousness measure. The original face-consciousness items (X. Zhang, 2012) were designed to measure dispositional face consciousness; answered from participants’ own general perspective, they would largely register pre-existing face consciousness and thus fail to connect with the reading material about the image-transgressing sneakers. To establish a face-consciousness link between participants and the target product, the items were adapted to the TANIC context. In adapting the scale, we preserved its original two-dimensional structure—the desire to gain face and the fear of losing face—while contextualizing the items to the target product; this follows the established practice in consumer research of adapting dispositional scales to product-specific contexts. For example, “I try my best to hide my shortcomings so that others do not learn of them” was revised to “I try my best to hide the shortcomings of TANIC sneakers so that others do not learn of them”; “I care a great deal about others’ compliments and praise of me” was changed to “Owning TANIC sneakers can earn me compliments and praise from others”; and “If my school is not a good one, I try not to mention it to others” was changed to “If the TANIC sneakers I bought are not good, I try not to mention them to others.” The adapted five-item questionnaire was used in this experiment. To validate the adaptation, 122 participants completed a pretest of the adapted questionnaire, which showed high internal consistency (α = 0.874). To provide additional evidence of construct validity, we conducted a confirmatory factor analysis (CFA) on these pretest data. Based on modification indices, two residual covariances (between items 1 and 2 and between items 4 and 5) were freed, reflecting shared variance among items capturing the desire to gain face and the fear of losing face, respectively. The model demonstrated good fit, χ^2^(3) = 4.741, *p* = 0.192; CFI = 0.995; TLI = 0.983; RMSEA = 0.069 (90% CI [0.000, 0.180]); SRMR = 0.018, with standardized factor loadings ranging from 0.59 to 0.94 (all *p*s < 0.001). These results provide evidence that the adapted items adequately capture the intended face-consciousness construct despite the product-contextualization.

Perceived-risk measures. Perceived quality risk was measured with a five-item scale (e.g., “Purchasing this brand’s products could result in a substantial financial loss”; α = 0.872) and perceived psychological risk with a four-item scale (e.g., “The thought of purchasing this brand’s products makes me feel psychologically uncomfortable”; α = 0.922) (Laroche et al., 2004).

### 8.2. Results

Manipulation check. Participants in the image-transgression condition (*M* = 3.42, *SD* = 1.08) reported lower self-image congruence than those in the no-transgression condition (*M* = 3.77, *SD* = 1.04), *t*(258) = 2.58, *p* < 0.05, indicating a successful manipulation.

**Figure 3:**
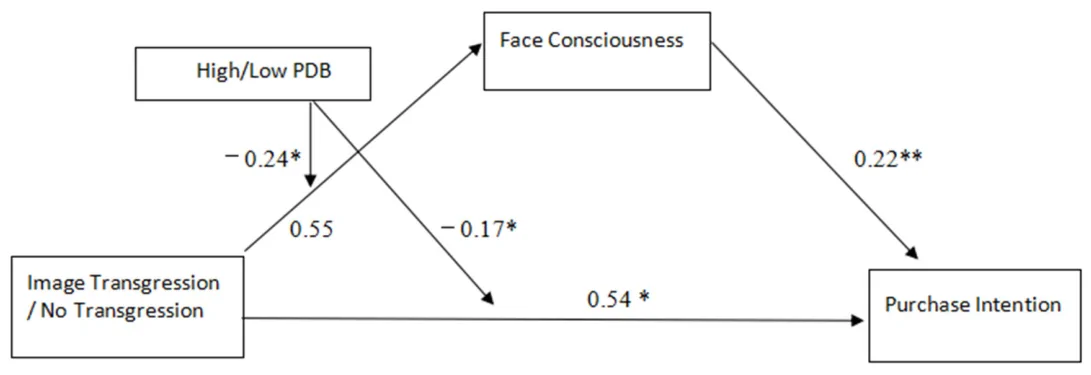
The mediating effect of face consciousness (Experiment 4). * *p* < 0.05, ** *p* < 0.001.

The mediating role of face consciousness. Power distance belief was significantly positively correlated with face consciousness (*r* = 0.38, *p* < 0.01): the higher the power distance belief, the stronger the face consciousness. A bootstrap analysis was then conducted with transgression condition as the independent variable, purchase intention as the dependent variable, face consciousness as the mediator, and power distance belief as the moderator, using PROCESS Model 8 with 5000 resamples and 95% confidence intervals (Hayes, 2013). The interaction between image transgression and power distance belief significantly predicted face consciousness (β = −0.24, *SE* = 0.10, 95% CI [−0.441, −0.046]): when confronted with an image transgression, consumers with higher power distance belief exhibited stronger face consciousness. The interaction also significantly predicted purchase intention (β = −0.17, *SE* = 0.08, 95% CI [−0.334, −0.014]): when confronted with an image transgression, consumers with higher power distance belief showed lower purchase intention. The interaction of image transgression and power distance belief indirectly influenced purchase intention through face consciousness, and the index of moderated mediation was significant (Index = −0.05, *SE* = 0.02, 95% CI [−0.11, −0.0014]); see Figure 3.

Mediation analyses of perceived risk. A parallel test with PROCESS Model 8 (5000 resamples), with perceived quality risk as the mediator and power distance belief as the moderator, found no mediating effect (Index = −0.02, *SE* = 0.02, 95% CI [−0.08, 0.01]). Likewise, an analysis with perceived psychological risk as the mediator showed no mediating effect (Index = −0.01, *SE* = 0.02, 95% CI [−0.05, 0.01]).

Going further, face consciousness and perceived quality risk were entered simultaneously as parallel mediators, with power distance belief as the moderator. The mediating effect through face consciousness remained significant (Index = −0.05, *SE* = 0.02, 95% CI [−0.11, −0.0017]), whereas the effect through perceived quality risk was not significant (Index = −0.009, *SE* = 0.02, 95% CI [−0.06, 0.02]). Similarly, with face consciousness and perceived psychological risk as parallel mediators, the mediating effect through face consciousness was significant (Index = −0.06, *SE* = 0.03, 95% CI [−0.13, −0.003]) but the effect through perceived psychological risk was not (Index = 0.01, *SE* = 0.02, 95% CI [−0.02, 0.05]).

### 8.3. Discussion

The results support H3: under image transgression (vs. no transgression), high-PDB consumers experience stronger face consciousness, which in turn is associated with a more pronounced decline in purchase intention than that of low-PDB consumers. The findings provide evidence consistent with face consciousness playing an important mediating role in the process by which power distance belief and image transgression jointly influence purchase intention: because of the incongruence between the brand image and their self-image, high-PDB consumers—owing to their stronger face consciousness—experience a more pronounced decline in purchase intention. This extends face consciousness from its established role as an antecedent of conspicuous and status consumption to that of an important mediating mechanism underlying negative consumption decisions in brand-transgression contexts. We note, however, that in this experiment purchase intention was measured before face consciousness. Although this measurement order does not invalidate the statistical analysis, it renders the causal interpretation of the mediation less straightforward, and the present results should therefore be read as evidence consistent with—rather than definitive proof of—the proposed mediating process. Alternative accounts, such as social identity threat, self-brand disidentification, status threat, perceived loss of exclusivity, in-group/out-group distinction, and the need for uniqueness, remain plausible and warrant examination in future research.

Notably, in Experiment 4 the competitive parallel mediation tests provided no significant support for a mediating role of perceived quality risk or perceived psychological risk in this relationship, although both are mediators identified in prior research on quality transgression (Huang & Deng, 2022). Under image transgression, the present data are therefore more consistent with face consciousness than with perceived risk as the mechanism underlying the purchase responses of consumers with different power distance beliefs. This result points to a difference between the psychological mechanisms of the two transgression types: image transgression is in essence a “social-attribute” crisis whose core threat lies in damage to consumers’ social identity and face needs, rather than in concerns about product functionality or psychological comfort, whereas in the present data the perceived-risk mechanism commonly observed under quality transgression did not receive significant support for image transgression.

## 9. Experiment 5: The Moderating Role of Public Versus Private Contexts

Experiment 5 explored the boundary conditions of the effect of power distance belief under image transgression, testing H4: in public contexts, the decline in purchase intention toward an image-transgressing brand (vs. a non-transgressing brand) is more pronounced for high-PDB than for low-PDB consumers, whereas in private contexts the difference narrows and is expected to be no longer statistically significant. By introducing usage context as a situational variable, this experiment specifies the boundary within which the face-consciousness mechanism applies, thereby refining the theoretical framework of consumer decision making under image transgression.

### 9.1. Method

A 2 (power distance belief: high vs. low) × 2 (transgression: image transgression vs. no transgression) × 2 (usage context: public vs. private) between-subjects design was adopted. The required sample size was calculated with G*Power 3.1 (Faul et al., 2009) with effect size *f* = 0.40, α = 0.05, and power = 0.80, yielding a minimum required sample size of 86. Participants were recruited through relevant elective courses and received course credit after completing the questionnaire. A total of 277 participants were recruited, of whom 34 did not complete the scrambled-sentence priming task and the accompanying reason-writing task, 22 failed the reading-material check question, and 2 failed the attention-check question. The final valid sample comprised 219 participants (39% male; aged 18–23 years, *M*_age_ = 19.36, *SD* = 0.84).

Power distance belief was first primed following the procedure of Experiment 1, and participants completed the power distance belief items (Yoo et al., 2011) (α = 0.801). Participants then read the image-transgression (or no-transgression) material, identical to Experiment 2; the manipulation check employed the self-image congruence scale (Sirgy et al., 1997) (α = 0.90). Next, usage context was manipulated through purchase scenarios. In the private-context condition, participants read, “You see that these sneakers are on sale, and you happen to need a new pair of sneakers; you want to buy a pair to take home and wear when you go night running alone in the evening,” accompanied by a picture of a person night running alone. In the public-context condition, participants read, “You see that these sneakers are on sale, and you happen to need a new pair of sneakers; you want to buy a pair to take home and wear the next time you go out with your classmates for a class team-building activity,” accompanied by a picture of a class team-building outing. Finally, purchase intention (Huang & Deng, 2022) (α = 0.915) and demographic information were collected.

### 9.2. Results

Manipulation checks. Independent-samples *t* tests showed that participants in the high-PDB condition (*M* = 2.94, *SD* = 1.10) reported higher PDB than those in the low-PDB condition (*M* = 2.62, *SD* = 1.04), *t*(217) = 2.24, *p* < 0.05, indicating successful priming. Participants in the image-transgression condition (*M* = 3.36, *SD* = 1.19) scored lower on self-image congruence than those in the no-transgression condition (*M* = 3.75, *SD* = 1.26), *t*(217) = 2.29, *p* < 0.05, indicating a successful transgression manipulation.

Purchase intention. A three-way ANOVA was conducted with power distance belief (high vs. low), transgression condition (image transgression vs. no transgression), and usage context (public vs. private) as independent variables and purchase intention as the dependent variable. The interaction between power distance belief and transgression condition was significant, *F*(1, 211) = 4.10, *p* = 0.044, η_p_^2^ = 0.019: under high PDB, purchase intention was lower in the image-transgression condition (*M* = 4.37, *SD* = 1.45) than in the no-transgression condition (*M* = 4.56, *SD* = 1.09). The interaction between power distance belief and usage context was also significant, *F*(1, 211) = 14.07, *p* < 0.001, η_p_^2^ = 0.063: under high PDB, purchase intention was lower in the public context (*M* = 3.92, *SD* = 1.40) than in the private context (*M* = 4.96, *SD* = 0.96). The interaction between transgression condition and usage context was not significant, *F*(1, 211) = 1.57, *p* = 0.21, η_p_^2^ = 0.007.

**Figure 4:**
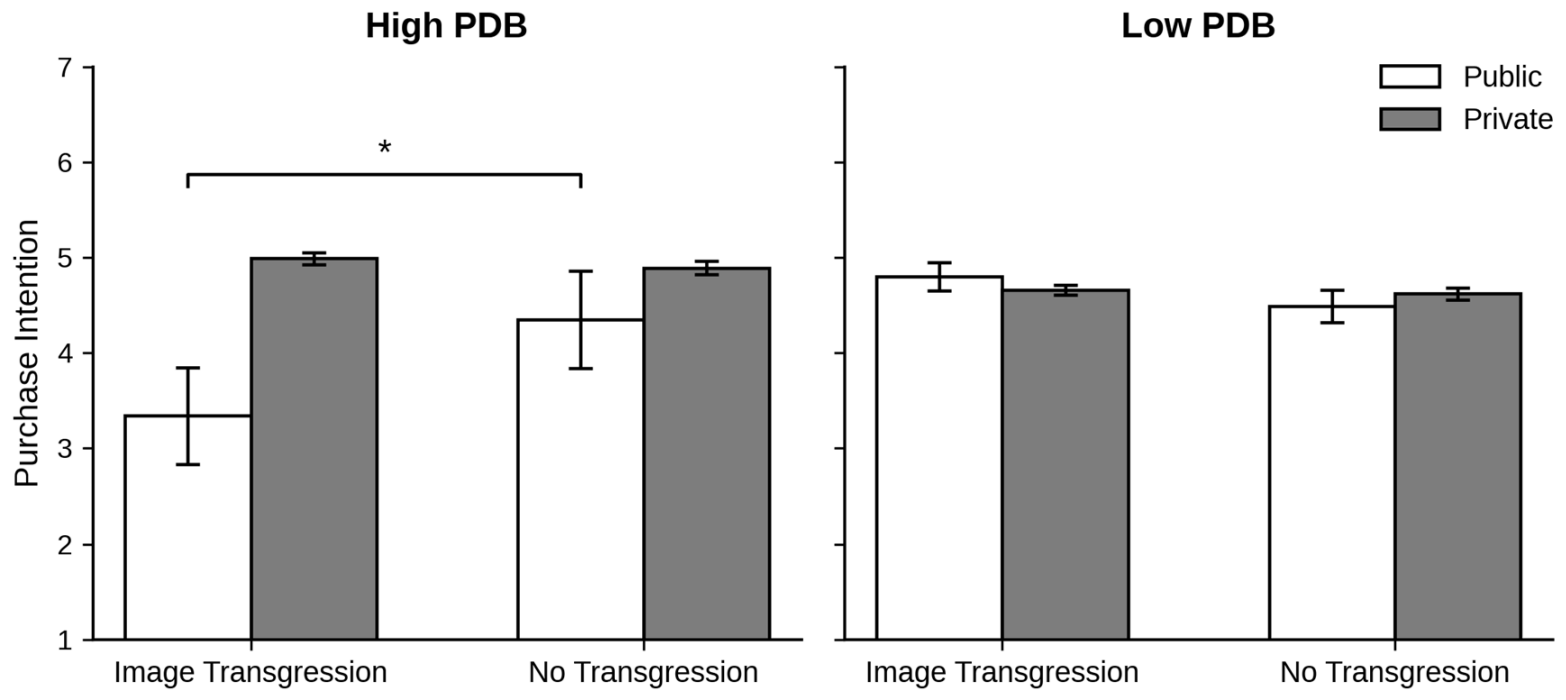
Purchase intention of consumers with different power distance beliefs under image transgression across usage contexts (Experiment 5). Error bars represent ±1 *SE*. * *p* < 0.05.

More importantly, the three-way interaction of power distance belief, transgression condition, and usage context was significant, *F*(1, 211) = 5.43, *p* = 0.021, η_p_^2^ = 0.025. When facing an image transgression, high-PDB participants reported markedly lower purchase intention in the public usage context (*M* = 3.34, *SD* = 1.57) than in the private context (*M* = 4.99, *SD* = 0.97), whereas low-PDB participants’ purchase intention did not differ noticeably between the public (*M* = 4.80, *SD* = 0.90) and private (*M* = 4.66, *SD* = 1.22) contexts (see Figure 4). H4 was thus supported.

Conditional interactions and simple effects. To clarify the significant three-way interaction, we examined the power distance belief × transgression interaction separately within each usage context. In the public context, the interaction was significant, *F*(1, 96) = 8.40, *p* = 0.005. Within the image-transgression condition, high-PDB participants reported significantly lower purchase intention (*M* = 3.35, *SD* = 1.57, *n* = 23) than low-PDB participants (*M* = 4.80, *SD* = 0.90, *n* = 23), mean difference = 1.46, 95% *CI* [0.80, 2.12], *p* < 0.001. Within the no-transgression condition, high-PDB (*M* = 4.34, *SD* = 1.12, *n* = 32) and low-PDB (*M* = 4.48, *SD* = 0.73, *n* = 22) participants did not differ significantly, mean difference = 0.13, 95% *CI* [−0.49, 0.75], *p* = 0.670. Examined from the alternative direction, high-PDB participants reported significantly lower purchase intention under image transgression (*M* = 3.35, *SD* = 1.57, *n* = 23) than under no transgression (*M* = 4.34, *SD* = 1.12, *n* = 32), mean difference = 1.00, 95% *CI* [0.38, 1.61], *p* = 0.002, whereas low-PDB participants showed no significant difference between the image-transgression (*M* = 4.80, *SD* = 0.90, *n* = 23) and no-transgression (*M* = 4.48, *SD* = 0.73, *n* = 22) conditions, mean difference = −0.33, 95% *CI* [−0.99, 0.34], *p* = 0.333. In the private context, the interaction was not statistically significant, *F*(1, 115) = 0.05, *p* = 0.819, indicating that the moderating role of power distance belief in responses to image transgression was attenuated in private usage settings. High-PDB participants did not differ significantly between the image-transgression (*M* = 4.99, *SD* = 0.97, *n* = 38) and no-transgression (*M* = 4.90, *SD* = 0.98, *n* = 20) conditions, and low-PDB participants likewise showed no significant difference between conditions (*M* = 4.60, *SD* = 1.22, *n* = 39 for image transgression; *M* = 4.60, *SD* = 0.94, *n* = 22 for no transgression). H4 was therefore supported.

### 9.3. Discussion

Using a three-factor between-subjects design, Experiment 5 examined the boundary role of public versus private usage context with respect to the effect of power distance belief under image transgression. The significant three-way interaction was consistent with H4: under brand image transgression, high-PDB consumers’ purchase intention was strongly affected by the usage context—dropping significantly in public usage contexts—whereas low-PDB consumers’ purchase intention was unaffected by the usage context. This pattern is consistent with the face-consciousness mechanism: the core feature of face consciousness lies in obtaining social approval through others’ evaluations. Public contexts amplify social-evaluation pressure, intensifying high-PDB consumers’ sense of face loss and thereby driving a larger decline in purchase intention; in private contexts, the absence of external evaluative pressure reduces the activation of face consciousness, and the moderating effect of power distance belief accordingly weakens and is no longer statistically significant. We caution, however, that the public and private conditions differed in more than the observability of the product: the private condition involved running alone at night, whereas the public condition involved a class team-building activity with classmates. The two scenarios therefore also differed in social presence, peer evaluation, group context, interpersonal interaction, and activity type. Because social evaluation is itself closely related to the face-consciousness mechanism examined here, the stronger effect observed in the public condition may have arisen from heightened peer evaluation or from the broader social context rather than from visibility per se. We therefore characterize the Experiment 5 finding as a broader public-versus-private social-context effect and interpret the role of usage-context visibility with caution. We further note that the manipulation check collected in this experiment was a scenario-comprehension/recall check confirming that participants correctly understood and recalled their assigned usage scenario, rather than a direct measure of perceived visibility.

From the perspective of boundary conditions, Experiment 5 further consolidates the theoretical framework of this research. On the one hand, the moderating effect of usage context specifies the situations in which the face-consciousness mechanism applies—the mechanism is more likely to operate when consumption takes place in a public, socially evaluative setting—which is consistent with the theoretical positioning of face consciousness as a concern for social image. As cautioned above, however, the public and private scenarios also differed in social presence and activity type; we therefore interpret this boundary as a broader public-versus-private social-context effect rather than as evidence that visibility alone is decisive. On the other hand, the result carries a clear strategic implication for brand marketing practice: when a brand commits an image transgression, the risk of losing high-PDB consumers is especially pronounced in public consumption settings.

## 10. General Discussion

### 10.1. Summary of Findings

This research investigated the effect of power distance belief on consumers’ purchase intention under brand image transgression and explored the underlying mechanism and boundary conditions of this effect. Through the manipulation or measurement of consumers’ power distance beliefs, five progressively structured experiments revealed how purchase intention changes under different power distance beliefs and the role that face consciousness plays in this process, forming a converging body of evidence. The core conclusions can be summarized in four points. It is important to be explicit about the distinct evidentiary role of each experiment: Experiment 3a is correlational; Experiment 3b experimentally manipulates PDB but does not include brand image transgression; and Experiment 4 measures rather than manipulates PDB and measures face consciousness rather than manipulating it experimentally. The five experiments therefore provide converging evidence for a proposed causal model rather than a single, fully integrated experimental test of an established causal chain. It is also of note that the pattern is not confined to studies in which PDB was primed: Experiments 3a and 4 both operationalized PDB as a measured individual difference, with no priming procedure involved, and produced the same pattern of results. Because no priming task was administered in those studies, this convergence argues against the possibility that the present findings reflect features specific to the hierarchy/status-salience priming procedure rather than PDB itself.

First, robust verification of the basic effect. Under brand image transgression (vs. no transgression), the decline in purchase intention among high-PDB consumers was significantly greater than that among low-PDB consumers. This effect remained stable after controlling for confounds such as price promotion, verifying that power distance belief is a core moderating variable shaping the intensity of consumers’ reactions to image transgression.

Second, causal testing of the antecedent relationship. By combining correlational measurement with experimental manipulation, the causal relationship between power distance belief and face consciousness was established—high power distance belief strengthens face consciousness—laying a solid premise for the mediation tests.

Third, evidence for the mediating pathway. Through a moderated mediation model and competitive parallel-mediation tests, the findings were consistent with face consciousness playing an important mediating role in the interactive effect of power distance belief and image transgression on purchase intention. The decline in high-PDB consumers’ purchase intention under image transgression was associated with damage to their face consciousness, whereas perceived quality risk and perceived psychological risk—mediators commonly observed in quality-transgression contexts—did not receive significant support in the present data, suggesting a mechanistic boundary between transgression types.

Fourth, delineation of the boundary condition. The three-way interaction test indicated that usage context moderates the effect of power distance belief: in public usage contexts, the decline in purchase intention toward an image-transgressing brand was significantly greater for high-PDB consumers than for low-PDB consumers, whereas in private usage contexts the difference between the two groups was not statistically significant. Because the public and private scenarios also differed in social presence and activity type, we interpret this boundary as reflecting a broader public-versus-private social-context effect rather than visibility per se.

Together, Experiments 1–5 provide converging evidence for a proposed theoretical framework linking power distance belief, an important psychological mechanism, and a boundary condition in consumer responses to image transgression, specifying the pathway through which power distance belief influences purchase intention via face consciousness and the moderating effect of public versus private usage context.

### 10.2. Theoretical Contributions

This research makes several theoretical contributions.

First, it extends the study of power distance belief into the domain of brand transgression—and specifically into image transgression—thereby enriching both studies. Prior research has shown that PDB affects impulsive buying (Y. Zhang et al., 2010), charitable behavior (Winterich & Zhang, 2014), and brand preferences based on design origin (Paharia & Swaminathan, 2019), among other outcomes. With respect to brand transgression, existing work has focused on quality and moral transgressions—for example, consumers lower in power distance belief empathize more with victims of corporate moral transgressions, which heightens harm perceptions and negative moral emotions and produces unfavorable responses toward the transgressor (Xu et al., 2021)—but has not yet addressed the influence of PDB under image transgression. By verifying the effect of PDB on purchase intention under brand image transgression and its underlying psychological mechanism, this research extends the reach of both the PDB literature and the brand-transgression literature.

Second, the finding that high-PDB consumers’ purchase intention declines more sharply under image transgression directly extends the classic proposition linking power distance belief to preferences for status symbols. Hofstede (2001) defined power distance as the extent to which individuals accept the unequal distribution of power in society, and subsequent consumer research has found that individuals high in power distance generally have a stronger motivation for status differentiation and are more inclined to signal their social rank by consuming goods with socially conspicuous attributes (Gao et al., 2016). This research extends that conclusion from positive consumption choice to negative consumption rejection: when a brand’s attributes as a status symbol are undermined by its adoption among a disliked group, high-power-distance consumers actively sever their association with that status symbol by forgoing purchase—an extension of the status-differentiation motive into a negative context. Whether in positive choice or negative rejection, the decision core of high-power-distance consumers is to maintain the fit between their social identity and their status symbols.

Third, this research broadens the scope of research on self-image congruence and face consciousness. Consumer researchers have long drawn on self-image congruence to explain and predict product choice, brand choice, brand attitudes, purchase intention, and store loyalty and preference (Sirgy et al., 1997). Prior research has examined the roles of self-image congruence and face consciousness in consumers’ smartphone purchase decisions (Kaur & Soch, 2018), but it neither probed the underlying factor of power distance belief nor revealed why some consumers care more about face than others. Through experimental manipulation and mediation analyses, the present research provides evidence that PDB is an important antecedent of face consciousness: the face needs of high-PDB consumers are, in essence, a defense of the social hierarchical order rather than a mere pursuit of personal reputation. This finding advances face-consciousness research from phenomenon description toward mechanism explanation and enriches face theory in cultural psychology.

Fourth, the finding that face consciousness mediates the relationship between PDB and purchase intention under image transgression complements the literature on the mediating pathways linking power distance to consumption decisions. Prior work has proposed self-control as the mediator through which power distance influences impulsive consumption (Y. Zhang et al., 2010) and has emphasized prestige and status motives in face-related consumption (J. J. Li & Su, 2007), but the mediating role of face consciousness under image transgression had rarely been examined. This research provides convergent evidence for the mediating pathway across progressive experiments, supports its robustness using bootstrap methods, and shows that high-PDB individuals treat brands as symbolic carriers of social hierarchy—outward expressions of their social status and rank (Gao et al., 2016). When a brand’s image is transgressed, high-PDB consumers experience the change as a devaluation of their own social identity, which triggers a strong sense of face loss and ultimately a sharp decline in purchase intention.

Fifth, this research found that the mediating effect of perceived risk—the mediator identified in prior research on quality transgression and product-harm crises (Cleeren et al., 2017; Huang & Deng, 2022)—was not statistically significant. Using competing-mediator models, we found that under image transgression, the mediating effects of perceived quality risk and perceived psychological risk were not significant, whereas face consciousness remained a significant mediator in the same models. This result suggests a mechanistic boundary between transgression types: image transgression constitutes a “social-attribute” crisis whose core threat targets consumers’ social identity, whereas quality transgression constitutes a “functional-attribute” crisis whose core threat targets consumers’ safety needs—the two appear to rest on different mediating mechanisms.

Finally, this research clarifies the higher-order boundary effect of usage context, refining the contextual conditions under which power distance belief operates. Prior research on power distance and public consumption has focused mostly on premium effects in positive consumption scenarios—Chinese consumers are willing to pay more when products are used in public settings (Lin et al., 2013), and high-PDB consumers show elevated purchase intention only for publicly displayable luxury categories (Gao et al., 2016)—implicitly assuming that public settings only reinforce positive consumption decisions. This research extends the logic of social display to the negative scenario of brand transgression and shows that usage context acts as a higher-order moderator determining whether the core effect holds: the moderating effect of PDB emerged in the public, socially evaluative consumption context—in which an image-damaged brand conveys the negative signal that “the user has chosen a brand inconsistent with their identity”—but not in the private one. In private contexts, the social dimension of consumption recedes, and the effect of brand transgression on purchase intention was not statistically significant regardless of the individual’s power distance level. This finding fills a gap concerning the operation of public versus private contexts in negative consumption scenarios. Because the public and private scenarios also differed in social presence and activity type, we frame this contribution in terms of public versus private social contexts rather than of observability per se.

### 10.3. Managerial Implications

The findings offer several implications for marketing practice.

First, firms should attach greater importance to brand transgression—particularly the influence of image transgression on consumer behavior. As consumption levels rise and choices multiply, consumer needs grow increasingly complex and tolerance for brand transgressions keeps declining. Meanwhile, the rapid development of online media platforms makes information ever easier to spread: anyone can become an information publisher, and consumer complaints and negative brand information proliferate across the Internet. Mishandling a transgression can therefore have severe consequences, directly affecting sales and reputation and, in extreme cases, threatening the firm’s survival. Quality and moral transgressions typically cause direct, visible losses and thus receive managerial attention; brand image transgression is more covert, yet it likewise inflicts real losses and can cost a firm large numbers of existing customers. The present results show that under the form of image transgression examined here—brand-image dilution involving loss of exclusivity and association with disliked users—the purchase intention of high-PDB consumers declines significantly. Firms should therefore attend, from the source, to the congruence between brand products and consumers’ images and avoid image transgression as far as possible.

Second, the results can guide firms in formulating marketing policies appropriate to consumers holding different power distance beliefs—especially under economic pressure. When cash flow is tight, or products are slow-moving, merchants often resort to promotional discounting. Although discounting can raise sales and reduce inventory, it carries risks: when large numbers of non-target customers begin to purchase a brand’s products, the image the brand has cultivated may collapse or even reverse, and the original consumer base may defect after seeing consumers whose images are inconsistent with their own using the product. Firms should weigh image-transgression risk when designing promotions: a brand’s core flagship products should not be subjected to large-scale markdown promotions, lest the same product flood the streets and damage the social image of the existing customer base; when facing economic pressure, firms can instead adopt stored-value (top-up) promotions that encourage the existing customer base to keep purchasing and strengthen brand identification. Particular attention should be paid to the face consciousness of high-PDB consumer groups: when targeting high-PDB consumers, firms can adopt marketing strategies that affirm face, enabling these consumers to identify strongly with their own image during the purchase process and strengthening the congruence between their image and the brand’s image.

Third, the results help firms recover from, and guard against, the consequences of image transgression. Under image transgression, high-PDB consumers show lower purchase intention because their face consciousness has been damaged; firms can retain the original consumer base by restoring the face consciousness of high-PDB consumers, and only with the correct recovery approach can consumers be induced to repurchase the brand. Brand image is not merely the outward manifestation of a firm’s products or services but also consumers’ cognitive and emotional experience of the firm, and power distance belief, as an individual-level cultural value, profoundly shapes consumers’ perception and acceptance of brand image. When reshaping and restoring a damaged brand image, firms should fully consider the needs and expectations of consumers with different power distance beliefs. For high-PDB consumers, the restored brand image should emphasize symbols of premium positioning, distinctiveness, and social status—for example, by associating the brand with high-end culture, the arts, or social events to elevate the brand’s image value in these consumers’ minds.

### 10.4. Limitations and Future Research

This research validated its conclusions through five progressively structured experiments and carries significance at both the theoretical and practical levels. Nevertheless, several limitations should be acknowledged.

First, the sample population was limited to Chinese university students (ages 18–23), which constrains the generalizability of the findings to older consumers, working professionals, high-income individuals, and consumers at different status levels, none of which were represented in our samples. Although the present research employed both chronic measurement and experimental priming of PDB, the priming paradigm captures situational activation rather than the deeply internalized cultural orientations that characterize genuine cross-cultural differences. The core mechanism of face consciousness, moreover, is deeply embedded in East Asian cultural contexts; whether the same moderated mediation pathway holds in low-PDB cultural contexts (e.g., Northern European or Anglo-Saxon societies) remains an open question. Future research should replicate the model using more diverse samples—including participants of different ages, occupations, income levels, and status levels—and cross-cultural samples that differ in national-level power distance (e.g., China or Mexico vs. Germany or the Netherlands); because priming PDB captures situational activation rather than deeply internalized cultural orientations, replication in genuine cross-cultural contexts would be especially valuable.

Second, the reliance on a fictitious brand (TANIC) and scenario-based experimental paradigms limits ecological validity. Fictitious brands effectively control for extraneous associations, but real-world brand image transgressions unfold in richer informational environments—including word-of-mouth, social media discourse, and prior brand relationships—that may moderate the observed effects. The scenario-based measures captured purchase intention rather than actual purchase behavior, introducing a potential attitude–behavior gap. Future research should employ real brands and field experiments to strengthen external validity.

Third, the study examined only one manifestation of image transgression—brand-image dilution involving loss of exclusivity and association with disliked users—and one mediating pathway (face consciousness). Image transgression can take other forms—such as brand dilution through excessive licensing, repositioning that alienates core customers, or associations with stigmatized groups—each of which may activate different psychological mechanisms. Additionally, alternative theoretical accounts remain unexplored: the sharper decline in purchase intention among high-PDB consumers could be driven by social identity threat, self-brand disidentification, status threat, perceived loss of exclusivity, a stronger tendency toward in-group/out-group distinction, or the need for uniqueness, rather than by face consciousness per se. Future research should systematically compare multiple image-transgression subtypes and incorporate a broader set of potential mediators to refine the theoretical model.

Fourth, the manipulation of usage context in Experiment 5 (public vs. private) varied more than the observability of the product: the private condition involved running alone at night, whereas the public condition involved a class team-building activity with classmates, so the two scenarios also differed in social presence, peer evaluation, group context, interpersonal interaction, and activity type. Because social evaluation is itself closely related to the face-consciousness mechanism examined here, the Experiment 5 result should be interpreted as a broader public-versus-private social-context effect rather than as evidence of a specific effect of visibility. Future research should employ a cleaner manipulation in which the underlying consumption situation is held as constant as possible while only the observability of the product varies, and should include a direct manipulation check of perceived visibility.

Fifth, the experiments that manipulated power distance belief (Experiments 1, 2, 3b, and 5) all relied on essentially the same priming procedure—a scrambled-sentence task combined with a reason-writing task. Although employing a consistent manipulation is reasonable for replication purposes and provides continuity across the studies, this reliance means that the observed effects could in principle reflect features specific to this particular hierarchy/status-salience priming procedure rather than PDB itself. Evidence based on an alternative manipulation or operationalization of PDB would help establish that the findings reflect PDB rather than characteristics of the priming procedure. In addition, none of the present studies provides a fully integrated experimental test of the proposed process; future research would benefit from a design that experimentally manipulates both PDB and brand image transgression, measures the proposed mediator, and subsequently measures the focal behavioral outcome. Finally, in Experiment 4, purchase intention was measured before face consciousness; although this ordering does not invalidate the statistical analysis, it renders the causal interpretation of the mediation less straightforward, and future research should measure the mediator and the focal outcome in an order that permits stronger causal inference. It is worth noting in this connection that the same pattern emerged in the two studies in which PDB was measured rather than primed (Experiments 3a and 4); because no priming procedure was involved in either study, this convergence argues against the possibility that the observed effects are an artifact of the priming task. We nonetheless regard a fully integrated experimental test as the necessary next step for this line of work, and we intend to conduct it as the centerpiece of a follow-up study. To make this commitment concrete, the planned study will manipulate PDB using a procedure different from the scrambled-sentence/reason-writing task employed in the present experiments, experimentally manipulate brand image transgression while holding brand familiarity and price information constant across conditions, measure the proposed mediator before the focal behavioral outcome, and be preregistered, with the sample size determined by an a priori power analysis targeting the PDB × transgression interaction.

## 11. Conclusions

Across five experiments, this research shows that consumers’ responses to brand image transgression depend systematically on their power distance belief. Under image transgression, high-PDB consumers reduce their purchase intention markedly more than low-PDB consumers; this differential response is consistent with face consciousness—for which power distance belief is an antecedent—as an important mediating mechanism, whereas the mediating effects of perceived quality risk and perceived psychological risk were not statistically significant in the present data; the pattern was also more pronounced in public usage contexts. Theoretically, these findings extend power distance belief research into the understudied domain of brand image transgression, provide evidence consistent with face consciousness as an important mediating mechanism, and delineate public versus private usage context as its boundary condition. Practically, they alert firms to the covert costs of image-damaging actions such as aggressive discounting of flagship products and provide culturally informed guidance for designing promotion and recovery strategies that protect the face of high-PDB customers.

## Figures and Tables

**Table 1:** Overview of the five experiments.

Experiment	Design	Valid *N*	PDB	Key Result
1	2 (PDB: high/low) × 2 (transgression: image/no), between subjects	271	Primed	Significant PDB × transgression interaction on purchase intention; preliminary support for H1
2	As Experiment 1, with discount-matched no-transgression material	115	Primed	H1 supported; the price-promotion alternative did not receive significant support
3a	Correlational survey	138	Measured	PDB positively correlated with face consciousness (*r* = 0.280)
3b	Single factor (PDB: high/low), between subjects	181	Primed	Priming high PDB increased face consciousness (H2)
4	Single factor (transgression: image/no), between subjects; PDB measured	260	Measured	Findings consistent with face consciousness mediating the interactive effect; perceived-risk pathways did not receive significant support (H3)
5	2 (PDB) × 2 (transgression) × 2 (context: public/private), between subjects	219	Primed	Effect present in public usage contexts; not statistically significant in private contexts (H4)

## Data Availability

The original contributions presented in this study are included in the article. Further inquiries can be directed to the corresponding author(s).

## Appendix A

### Appendix A.1. Image-Transgression Reading Material

TANIC is a sneaker brand deeply loved by young people, and it has consistently performed very well in product design, product quality, and other respects. Since serving last year as a dedicated advertising partner of the Winter Olympic Games, TANIC has further won the hearts of the Chinese people and become wildly popular across China. It can be said that the TANIC brand is on a par with the favorite sports brand you selected earlier. TANIC sneakers target the college student population, and wearing TANIC sneakers has become a trend among college students; you feel that being able to buy and wear a pair of TANIC sneakers fits your image well. However, amid the economic downturn, sales of TANIC’s products have also stalled. To adapt to the economic climate and increase sales, the TANIC brand prepared to make a strategic adjustment. After deliberation, the TANIC brand decided to increase product sales through discounts and price reductions. After this adjustment, sales of TANIC’s products boomed for a time. The styles and colors of TANIC sneakers have remained unchanged, but the substantial drop in price has led many people in everyday life to start wearing TANIC sneakers, and the TANIC brand can now be seen everywhere in the streets. You notice that the TANIC brand keeps spreading to more and more groups of people. On one occasion, on a high-speed train, you saw a group of middle-aged men and women who were loudly playing videos on their phones and who were also wearing TANIC sneakers; the people around them cast odd looks their way, and you yourself found this playing of videos out loud quite off-putting. On another occasion, you discovered that some idle street youths, whom you had previously very much disliked, had also begun wearing the TANIC brand on their feet. You cannot help but begin to doubt whether the TANIC brand can still represent you.

### Appendix A.2. No-Transgression Reading Material (Experiment 1)

TANIC is a sneaker brand deeply loved by young people, and it has consistently performed very well in product design, product quality, and other respects. Since serving last year as a dedicated advertising partner of the Winter Olympic Games, TANIC has further won the hearts of the Chinese people and become wildly popular across China. It can be said that the TANIC brand is on a par with the favorite sports brand you selected earlier. TANIC sneakers target the college student population, and wearing TANIC sneakers has become a trend among college students; you feel that being able to buy and wear a pair of TANIC sneakers suits you well.

### Appendix A.3. No-Transgression Reading Material with Matched Discount Information (Experiments 2, 4, and 5)

TANIC is a sneaker brand deeply loved by young people, and it has consistently performed very well in product design, product quality, and other respects. Since serving last year as a dedicated advertising partner of the Winter Olympic Games, TANIC has further won the hearts of the Chinese people and become wildly popular across China. It can be said that the TANIC brand is on a par with the favorite sports brand you selected earlier. TANIC sneakers target the college student population, and wearing TANIC sneakers has become a trend among college students; you feel that being able to buy and wear a pair of TANIC sneakers suits you well.

One day you hear that TANIC sneakers are on an end-of-season sale, so you decide to find some time after class to browse the store properly. You check your schedule and find that you have only one long class in the afternoon, so after class you can walk from the teaching building to the subway station and take the subway there. After class, you notice that the sunshine is quite pleasant today; the sun feels warm on your body. You take a breath of fresh air and, as you walk, mull over whether or not to buy sportswear. Before you know it, you have arrived at the subway station. As usual, you take out your card and swipe it to enter. Once inside, you quickly find your train. After boarding, you check the route: about a few stops will bring you to the transfer point, and then one more line will take you to your destination. The subway is not very crowded, and everyone is busy with their own affairs. You think you might as well look at your phone first, so you start using your phone.
